# Impact of Blood Type and Administration Timing on Therapeutic Outcomes of Immune Checkpoint Inhibitors for Patients with Lung Cancer in the Chinese Alpine Region

**DOI:** 10.3390/cancers18091469

**Published:** 2026-05-02

**Authors:** Meiling Zhang, Xin Zhang, Tie Lin, Bao Liu, Jingwei Hao, Ziyi Gao, Xiaoli Li, Meng Wang

**Affiliations:** 1 Department of Respiratory Medical Oncology, Harbin Medical University Cancer Hospital, Harbin 150081, China; meilingzhang@hrbmu.edu.cn (M.Z.); 601664@hrbmu.edu.cn (B.L.); jingweihao@hrbmu.edu.cn (J.H.); ziyigao@hrbmu.edu.cn (Z.G.); 2 Department of Respiratory Medicine, The Fourth Affiliated Hospital of Harbin Medical University, Harbin 150006, China; xinzhang@hrbmu.edu.cn; 3 Department of Surgery, The First Affiliated Hospital of Harbin Medical University, Harbin 150001, China; tielin@hrbmu.edu.cn

**Keywords:** immunotherapy, lung cancer, time-of-day administration, circadian rhythm, prognosis

## Abstract

Background: Although immune function and tumor biology follow circadian rhythms, it is unclear whether optimizing the timing of immunotherapy can enhance clinical outcomes. This study evaluated the association between time-of-day administration of immune checkpoint inhibitors (ICIs) and treatment efficacy, and explored whether blood type modifies ICI response in Chinese lung cancer patients living in an alpine region. Methods: We performed a retrospective analysis of 1247 Chinese lung cancer patients treated with first-line chemoimmunotherapy between January 2016 and March 2021 at Harbin Medical University Cancer Hospital (Cohort 1, *n* = 839) and the Fourth Affiliated Hospital of Harbin Medical University (Cohort 2, *n* = 408). Primary endpoints were overall survival (OS) and progression-free survival (PFS) estimated by the Kaplan–Meier method. Secondary endpoints included immune-related adverse events (irAEs), disease control rate (DCR), and objective response rate (ORR). Results: Patients treated before 13:00 had superior outcomes compared with those treated later, with longer mOS (34.3 vs. 22.0 months, *p* < 0.0001) and mPFS (16.7 vs. 12.7 months, *p* < 0.001). The ORR was significantly higher in the before 13:00 group of Cohort 1 (64.4% vs. 55%, *p* = 0.005). Additionally, blood type O was associated with a higher irAEs rate (35.6% vs. 20.1%, *p* < 0.01) and longer mOS (34.5 vs. 23.1 months, *p* < 0.0001) than non-O types. Conclusions: Time-of-day administration and blood type may influence ICI efficacy, supporting circadian-informed and patient-tailored immunotherapy. These findings support incorporating circadian timing and patient-specific factors into cancer immunotherapy strategies.

## Simple Summary

Chronotherapy, which examines the influence of circadian rhythms on immune function and tumor biology, presents a promising approach to optimizing the efficacy of immune checkpoint inhibitors (ICIs) in lung cancer treatment. In this article, we explore the finding that patients with lung cancer treated with ICI infusion before 13:00 had superior outcomes (*p* < 0.05) compared with those treated later. Among the analyzed subgroups by blood type, we discovered a novel insight that patients with blood type O derived greater survival benefits (*p* < 0.05) and a higher irAEs rate (*p* < 0.01) from ICI infusions. These findings may inform the future development of personalized chronotherapy by clinicians for treatment decision-making regarding ICI, with the potential to enhance therapeutic outcomes in patients with lung cancer.

## 1. Introduction

Despite major advances in diagnosis and treatment, lung cancer remains a leading cause of death worldwide. However, in China’s alpine region, lung cancer mortality is even higher, driven by elevated tobacco use and exposure to fine particulate matter (PM2.5) [1,2]. Recent evidence suggests that while immunotherapy has transformed lung cancer treatment, its effectiveness remains limited for many patients, largely due to variability in the immune system’s capacity to mount a tumor-specific response [3,4,5,6].

One of the key factors regulating the immune response is the body’s internal circadian rhythm [7]. The circadian rhythm plays a crucial role in regulating the timing of immune cell development and their migration into tissues [8]. Given the importance of circadian rhythms in regulating immune function, an increasing number of studies are focusing on how they play this role in the body. Several randomized trials have consistently shown that immune responses to vaccination vary with circadian timing, with morning vaccination eliciting stronger immunogenicity than evening vaccination for Bacillus Calmette–Guérin (BCG), hepatitis A, and influenza vaccines [9,10,11]. Similarly, in non-tumor models, myeloid cell-specific deletion of the core clock gene BMAL1 disrupted the normal rhythmic cycling of monocytes, leading to increased accumulation of inflammatory Ly6C-high monocytes [12]. Furthermore, in tumor models, mice inoculated with melanoma cells during the day exhibited higher dendritic cell (DC) counts and a stronger anti-tumor response, resulting in greater tumor suppression than those inoculated at night [13]. Researchers are increasingly investigating how circadian timing can enhance the effectiveness of tumor immunotherapy.

Programmed death 1 (PD-1) and its ligand (PD-L1) blockade remain the most extensively investigated immunotherapy for cancer treatment, particularly in lung cancer [14,15]. Few studies have shown that since PD-1 levels in immune cells vary throughout the day, the timing of immunotherapy administration may influence the strength of anti-tumor immunity [16,17,18,19]. In advanced melanoma, Qian et al. confirmed that the timing of cancer ICI infusion, which is influenced by the body’s circadian rhythm, significantly impacts treatment outcomes without associated adverse effects or toxicity [20]. However, in a pan-cancer analysis, van Rensburg et al. found no association between the timing of ICI administration and clinical outcomes [21]. This discrepancy between studies may be due to the immunogenic diversity of neoantigens, which determines how they are presented to and recognized by a patient’s T-cell receptor repertoire. Blood type is one of the genetic factors that influences the diversity of immunogenic neoantigens [22]. Compared with blood types A, B, or AB, type O blood tends to have a more diverse T-cell receptor repertoire [23,24]. However, the influence of blood type and the timing of immunotherapy administration on treatment outcomes in lung cancer remains underexplored.

It is important to consider that circadian variability may influence the early pharmacokinetics (PK) of ICIs [25]. In patients with solid tumors, anti-PD-1 agents such as pembrolizumab typically reach steady-state plasma concentrations after approximately 15 to 20 weeks of treatment, whereas anti-PD-L1 agents, such as atezolizumab, reach a plateau after approximately 9 to 12 weeks [26,27]. As the initial four treatment cycles occur entirely within this pre-steady-state phase, time-of-day-dependent differences in drug absorption, distribution, metabolism, or clearance are most likely to exert a clinically relevant impact during this early period. Consequently, circadian influences on ICI PKs may partially account for the observed differences in treatment efficacy based on the timing of drug administration. In this study, we aimed to assess the impact of ICI administration timing (morning versus afternoon) and ABO blood group on clinical outcomes in lung cancer patients receiving first-line chemoimmunotherapy.

## 2. Materials and Methods

### 2.1. Study Design and Participants

This study used a retrospective research design. Two cohorts of lung cancer patients treated with chemoimmunotherapy as first-line treatment were recruited from two hospitals. Cohort 1 consisted of patients who received treatment at Harbin Medical University Cancer Hospital between January 2016 and March 2021. Cohort 2 included patients who received treatment at the Fourth Affiliated Hospital of Harbin Medical University between January 2017 and March 2021.

The inclusion criteria were adults aged 18 years or older diagnosed with small cell lung cancer (SCLC), lung adenocarcinoma (LUAD), or lung squamous cell carcinoma (LUSC) who had received at least four cycles of standard first-line treatment combining an anti-PD-1/PD-L1 agent with chemotherapy. Patients were excluded if their pathologic type was unclear, if they had an Eastern Cooperative Oncology Group (ECOG) performance status greater than 1, a history of autoimmune disease, other primary tumors in different organs, driver alterations (EGFR, ALK, etc.), or if their medical records or survival information were incomplete, including missing data on the timing of PD-1/PD-L1 inhibitor infusion.

PD-L1 expression was assessed using clinically available IHC assays at each participating institution. The tumor proportion score (TPS) was used for evaluation, and expression was categorized as <1%, 1–49%, or ≥50%.

### 2.2. Procedures

The patient’s medical records were reviewed to extract all infusion times for treatment with anti-PD-1/PD-L1 agent, adverse events related to ICIs, and other clinical and pathological characteristics at the time of the first infusion of PD-1/PD-L1 inhibitor.

Anti-PD-1 ICIs, including pembrolizumab, tislelizumab, sintilimab, or camrelizumab, were administered at a fixed dose of 200 mg every 3 weeks. Anti-PD-L1 agents such as sugemalimab or atezolizumab were administered at a fixed dose of 1200 mg every 3 weeks. The PD-1/PD-L1 inhibitor was infused intravenously over 30 min, followed by standard chemotherapy protocols. LUAD was treated with pemetrexed plus carboplatin; LUSC was treated with paclitaxel and carboplatin, and SCLC was managed with etoposide and carboplatin.

We estimated the median time of day for treatment administration to be 13:10 (IQR, 12:11–14:22) in the pooled cohort (Table 1). This finding aligns with four previous studies that used 13:00 as a cut-off time in a study-level meta-analysis examining the impact of immunotherapy infusion timing on cancer survival [28]. Next, the infusion timing was analysed as a continuous variable, using Cox regression models incorporating periodic restricted cubic splines (RCS) to account for the periodic nature of infusion time and the risks of death and disease progression. RCS is a popular way to flexibly model non-linear relationships in regression models and is used to handle periodic data, such as infusion time [29]. Therefore, we adopted 13:00 as a biologically informed cut-off to define the onset of the afternoon.

Tumor response was assessed using computed tomography (CT), ultrasonography, magnetic resonance imaging (MRI), and/or positron emission tomography (PET). These images were generally acquired after the completion of every two treatment cycles. Tumor responses were classified according to the Response Evaluation Criteria in Solid Tumors (RECIST) version 1.1 as partial response (PR), complete response (CR), progressive disease (PD), or stable disease (SD) [30].

IrAEs related to treatment with ICIs were retrospectively assessed in the medical record from the start of treatment to the last follow-up. IrAEs were evaluated independently by two doctors according to the Common Terminology Criteria for Adverse Events (CTCAE) version 5.0 of the National Cancer Institute [31]. Generally, adverse events related to treatment with ICIs consist of autoimmune-mediated side effects, including the incidence of rash, colitis, hypothyroidism, liver dysfunction, and impaired glucose regulation.

### 2.3. Outcomes

The primary endpoints for this study were OS and PFS. OS was defined as the time from the first PD-1/PD-L1 inhibitor administration to all-cause death or the last follow-up visit for patients last seen alive. PFS was defined as the time from the first administration of a PD-1/PD-L1 inhibitor to death or disease progression (whichever occurred first) or the last follow-up visit for patients who remained alive without disease progression. The DCR was defined as the sum of PR, CR, and SD. The ORR was defined as the sum of CR and PR. The incidence of irAEs was recorded by clinicians during regular follow-up in line with the NCCN Guidelines on Immunotherapy-Related Toxicities, and was classified accordingly [32]. We coded irAE occurrence as 1 if any irAEs were reported at least once during the treatment course, and 0 if none were.

### 2.4. Statistical Analysis

Continuous variables were reported as medians and interquartile ranges (IQRs), and categorical variables were reported as frequencies and percentages. The clinical characteristics in each cohort were compared using the Chi-square (χ^2^) test for categorical variables and the Independent-Samples T Test for continuous variables. The RCS was used to assess the cut-off time. To formally test whether the effect of infusion timing on survival differed between blood type groups, we performed a multivariable Cox proportional hazards regression model including an interaction term between blood type and infusion timing. The interaction was considered statistically significant if the *p*-value for the interaction term was <0.05.

The ORR and DCR between the morning and afternoon treatment groups were compared using the χ^2^ test and the Bonferroni test. PFS and OS were estimated using the Kaplan–Meier method and compared using the log-rank test. The associations between the clinical characteristics of both cohorts combined and PFS and OS were computed using univariate and multivariable Cox proportional hazards regression. All statistical analyses were performed using SPSS Statistics (version 27, IBM Corporation, Armonk, NY, USA) and GraphPad Prism (version 10, GraphPad Software Inc., San Diego, CA, USA), and a *p*-value below 0.05 was considered statistically significant.

## 3. Results

### 3.1. Patient Enrollment and Baseline Characteristics

**Figure 1:**
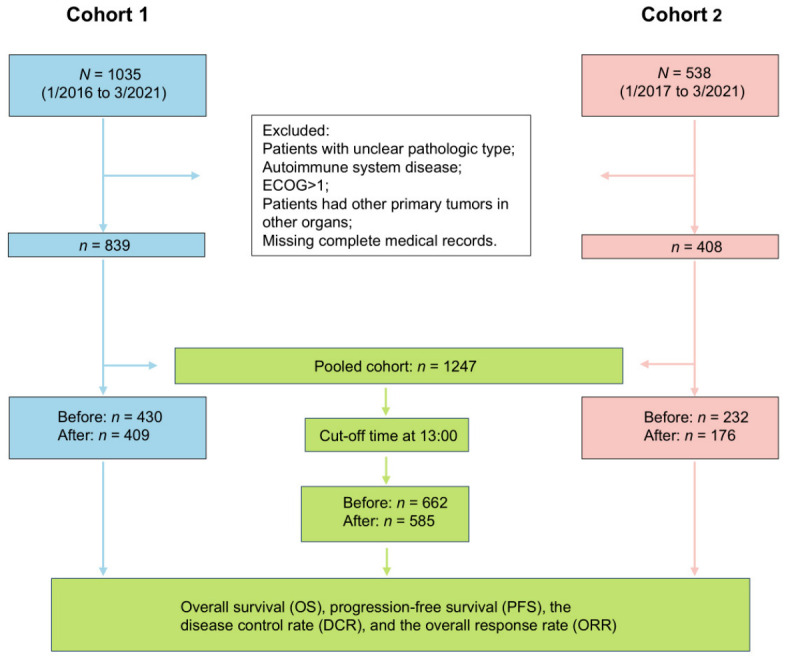
This flow chart illustrates the patients’ inclusion and exclusion criteria across all cohorts, detailing the study design and key endpoints in Cohort 1, Cohort 2, and the pooled cohort analysis. ECOG: Eastern Cooperative Oncology Group. Before: Before 13:00 group; After: After 13:00 group.

According to the inclusion and exclusion criteria, there were 839 out of 1035 patients (81.8%) in Cohort 1 and 408 out of 538 patients (75.8%) in Cohort 2 included. A total of 1573 patients were initially enrolled, and 1247 patients with lung cancer were ultimately included in the study (Cohort 1, *n* = 839; Cohort 2, *n* = 408). At the follow-up cutoff, 442 patients (33.8%) died (Figure 1).

Of the 1247 patients, 68.1% were male. The majority (55.7%) of the patients were either current or former smokers and presented with Stage IV (67.5%) metastatic disease. The most common metastatic sites were bone (31.6%), brain (21%), liver (15.1%), pleural or pericardial metastases (36.7%), and adrenal metastases (8.9%). PD-L1 expression was detected in 68.2% of the 872 patients tested. Patients were classified into three histological types: LUAD (42.7%), LUSC (24.6%), and SCLC (32.7%). More than half of the patients (58.1%) had blood type A, B, or AB (Table 1).

### 3.2. The Cut-Off Time for Daily Administration of Immunotherapy

**Figure 2:**
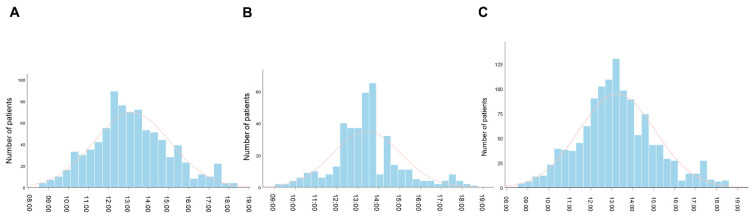
Distribution of per-patient median time-of-day of administration of ICI infusion onsets, for the initial four courses of immunotherapy. (**A**) Cohort 1; (**B**) Cohort 2; (**C**) Pooled Cohort.

The median time-of-day administration was 12:58 [IQR, 11:53–14:26] for Cohort 1 (Figure 2A), 13:20 [IQR, 12:31–14:14] for Cohort 2 (Figure 2B), and 13:10 [IQR, 12:11–14:22] for Pooled Cohort (Figure 2C).

**Figure 3:**
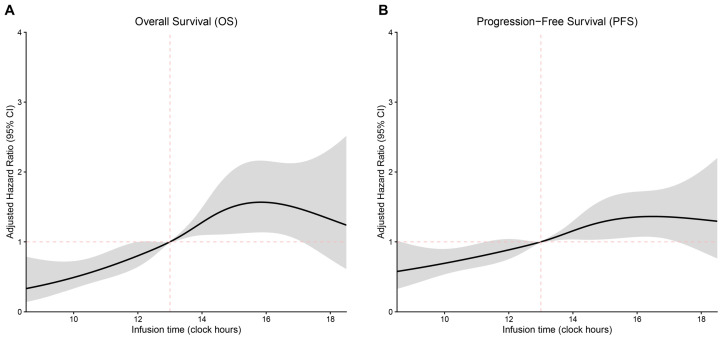
Multivariate Cox proportional hazards regression analysis of (**A**) OS and (**B**) PFS using RCS. The black lines represent the fitted curves of the association between time-of-day administration and the estimated hazard ratio (HR) after adjustment. The shaded areas represent the 95% confidence intervals (CIs). The models were adjusted for the following covariates: sex, age, smoking status, EOCG, tumor stage, PD-L1 expression, number of metastatic sites, and type of immunotherapy. The periodic RCS plot used the infusion time at 13:00 as the reference for HR calculation.

When time-of-day infusion was modeled as a continuous variable, afternoon administration was associated with a progressive increase in the risk of death and disease progression, whereas morning administration conferred a protective effect. The inflection point appeared to occur between 12:00 and 14:00 (Figure 3A,B).

### 3.3. The Median Survival Time Following the Administration of Immunotherapy

**Figure 4:**
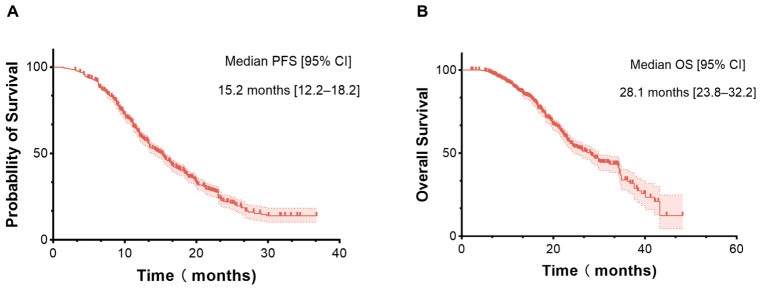
Overall efficacy of the immunotherapy protocols in the pooled cohorts. (**A**) Median progression-free survival; (**B**) Median overall survival.

The PFS curves of the whole cohort revealed a median PFS (mPFS) of 15.2 months (95% CIs 12.2–18.2) (Figure 4A). The OS curves of the whole cohort revealed a median OS (mOS) of 28.1 months (95% confidence intervals (CIs) 23.8–32.2) (Figure 4B).

### 3.4. Efficacy of Immunotherapy Administered “Before” or “After” 13:00

A total of 662 patients were assigned to the “before 13:00” group and 585 patients to the “after 13:00” group (Table 2). Significant differences were observed in blood type, smoking status, histological type, family history of cancer, and PD-L1 tumor proportion score covariates between patients in the two infusion groups. However, the two treatment groups showed no statistically significant differences in clinical and demographic features. Compared to the “before 13:00” group, the “after 13:00” group included a slightly higher proportion of patients with SCLC, more metastatic sites, a higher proportion of patients with type A/B/AB blood, and lower tumor PD-L1 expression. These factors may have contributed to worse outcomes in the “after 13:00” group. Additionally, the proportion of patients with adverse immune toxic reactions in the “after 13:00” group was higher, but the difference was not statistically significant.

**Figure 5:**
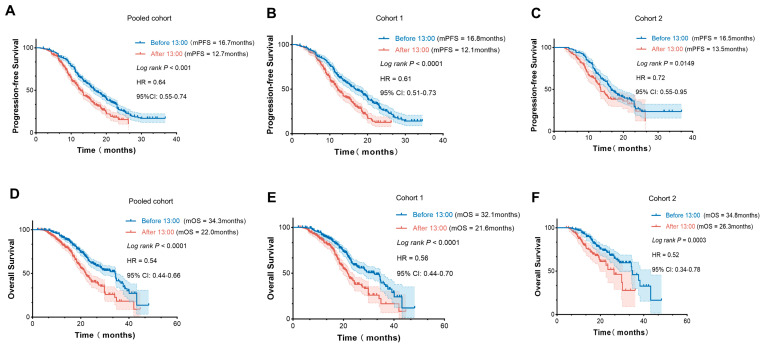
Treatment outcomes of patients with different time-of-day immunotherapy infusions. Kaplan–Meier curves analysis of PFS (**A**) and OS (**D**) in the pooled cohort; PFS (**B**) and OS (**E**) in Cohort 1; PFS (**C**) and OS (**F**) in Cohort 2. *P*-values were calculated using the log-rank test. HR: Hazard ratio; CI: Confidence interval; mOS: Median overall survival; mPFS: Median progression-free survival.

The Kaplan–Meier curves revealed statistically significant differences in both OS and PFS between patients treated before 13:00 and those treated after 13:00 (Figure 5). In the pooled cohort, mOS was 34.3 months in the “before 13:00” group, compared with 22.0 months in the “after 13:00” group [95% CI, 0.44–0.66] (*p* < 0.0001) (Figure 5D). The mPFS was 16.7 months vs. 12.7 months in the pooled cohort of the two infusion groups [95% CI, 0.53–0.74] (*p* < 0.0001) (Figure 5A). Notably, a similar trend between prognosis and injection timing for PD-1/PD-L1 inhibitors was observed in both Cohort 1 and Cohort 2 (Figure 5B,C,E,F).

Irrespective of histology or PD-L1 expression, the subgroup analysis showed that survival outcomes were significantly associated with the timing of ICI administration. In SCLC, median OS was 28.4 months in the “before 13:00” group versus 18.2 months in the “after 13:00” group (95% CI, 0.39–0.70; *p* < 0.0001) (Figure S1C), and median PFS was 14.3 versus 10.0 months, respectively (95% CI, 0.47–0.75; *p* < 0.0001) (Figure S1D). In non-small cell lung cancer (NSCLC), survival outcomes were similarly improved with morning infusions (*p* < 0.0001) (Figure S1A,B). Across all PD-L1 expression levels, prognosis was also better when ICI was administered before 13:00 (*p* < 0.05) (Figure S2).

**Figure 6:**
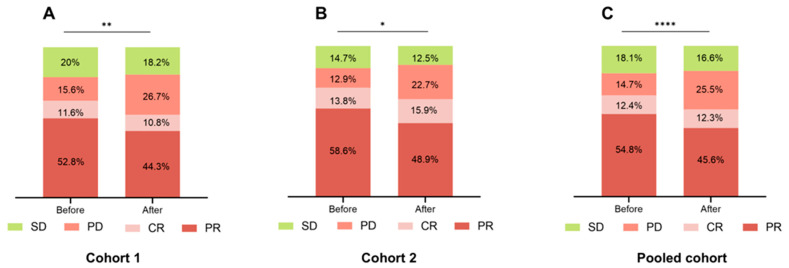
Response rates in each cohort’s “Before 13:00” and “After 13:00” groups. (**A**) Cohort 1; (**B**) Cohort 2; (**C**) Pooled cohort. (* *p* < 0.05, ** *p* <0.01, **** *p* <0.0001). PR: Partial response; CR: Complete response; PD: Progressive disease; SD: Stable disease; Before: Before 13:00; After: After 13:00.

Patients treated before 13:00 consistently showed a more favorable clinical response compared to those treated later in the day. In the pooled cohort, the “before 13:00” group had a significantly higher rate of PR (54.8% vs. 45.6%, *p* < 0.0001) and a lower rate of PD (14.7% vs. 25.5%, *p* < 0.0001). SD was also slightly more frequent in this group (18.1% vs. 16.6%, *p* < 0.0001), while CR remained similar between groups (12.4% vs. 12.3%, *p* < 0.0001). These trends were consistent across Cohort 1 and Cohort 2, where the “before 13:00” group again exhibited higher PR and SD rates and lower PD rates (Figure 6A–C).

Moreover, the correlation analysis of the clinical response rates with time-of-day administration revealed that the DCR was also higher in the “before 13:00” group in all Cohorts. However, following Bonferroni correction, none of these differences reached statistical significance. The ORR was significantly higher in the “before 13:00” group in Cohort 1 (*p* = 0.005) and the pooled cohort (*p* < 0.01), not in Cohort 2 (*p* = 0.098). Only the Cohort 1 ORR difference remained significant after Bonferroni correction (* in Table 3). Subgroup analyses revealed that blood type was consistently associated with DCR across all three cohorts, while smoking status showed a significant association with DCR in the pooled cohort. Both factors were also associated with ORR in the pooled cohort (Tables S1 and S2).

### 3.5. Univariate and Multivariate Survival Analyses of PFS and OS

**Figure 7:**
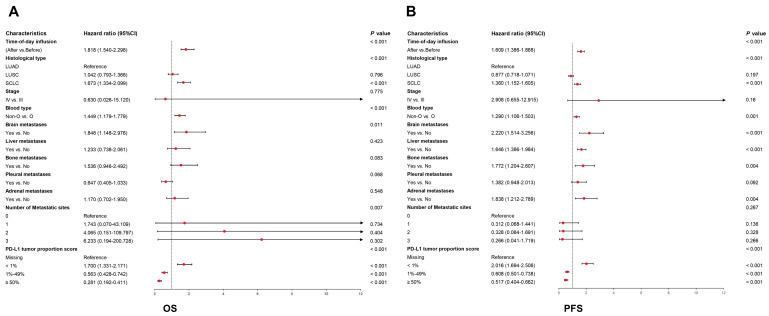
Multivariate Cox regression analyses of factors associated with OS (**A**) and PFS (**B**) in the pooled cohort. The red dots represent the point estimates of HR. CI: Confidence interval; HR: Hazard ratio; IrAEs: Immune-related adverse events.

In the pooled cohort, the univariate Cox regression analysis showed that time-of-day infusion, histological type, blood type, stage, PD-L1 tumor proportion score, and metastases were significantly associated with PFS and OS (Table S3). In addition, the time-of-day infusion (*p* < 0.001), blood type (*p* < 0.001), brain metastases (*p* = 0.011), histological type (*p* < 0.001), and PD-L1 tumor proportion score (*p* < 0.001) significantly affected OS (Figure 7A). In parallel, PFS was significantly associated with time-of-day infusion (*p* < 0.001), blood type (*p* = 0.001), histological type (*p* < 0.001), brain metastases (*p* < 0.001), liver metastases (*p* = 0.013), bone metastases (*p* = 0.004), adrenal metastases (*p* = 0.004), and PD-L1 tumor proportion score (*p* < 0.001) (Figure 7B). Cox regression analysis further confirmed the advantage of PD-1/PD-L1 inhibitor infusion before 13:00 for OS and PFS. These results demonstrated that time-of-day infusion was an independent prognostic factor for PFS and OS in patients who accepted PD-L1/PD-1 inhibitors.

### 3.6. Association of the Stratified Blood Type with the Infusion of ICIs and irAEs

Patients with the O blood type were significantly more likely to experience at least one irAE compared to those with blood types A, B, or AB (35.6% versus 20.1%, *p* < 0.001). Univariate logistic regression analysis showed that patients with blood types A, B, or AB had a significantly lower likelihood of developing irAEs relative to those with type O blood (odds ratio [OR]: 0.481; 95% CIs: 0.374–0.620; *p* < 0.01). Multivariate logistic regression further identified blood type as an independent and significant prognostic factor for irAEs, with a higher risk observed in type O patients compared to those with A, B, or AB blood types (A/B/AB vs. O: OR = 0.486; 95% CI: 0.377–0.627; *p* < 0.01) (Table 4).

**Figure 8:**
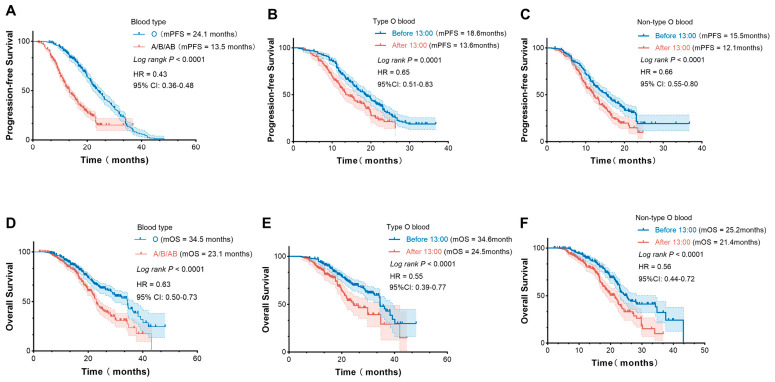
Treatment outcomes of PFS. (**A**) and OS (**D**) following the administration of immunotherapy, type O blood compared with type A/B/AB blood; Treatment outcomes of patients with different time-of-day immunotherapy infusions, Kaplan–Meier curves analysis of type O blood (**B**,**E**); The A/B/AB type blood (**C**,**F**). *p*-values were calculated using the log-rank test. CI: Confidence interval; HR: Hazard ratio.

Patients with type O blood had better OS and PFS than those with non-O blood in the pooled cohort (Figure 8A,D). Figure 8B,E show the survival of type O blood patients, stratified by the timing of ICIs. Figure 8C,F show the survival of non-O blood type patients, stratified by the timing of ICIs. Across the entire cohort, participants receiving infusions before 13:00 yielded significantly longer PFS and OS than those receiving infusions after 13:00. However, patients with the O blood type achieved superior PFS (18.6 months vs. 15.5 months) and OS (34.6 months vs. 25.2 months) compared with non-O blood type patients (*p* < 0.05). In addition, we conducted a subgroup analysis stratified by TPS in NSCLC. Regardless of the TPS expression status, patients with type O blood had a better prognosis than those with non-type O blood (*p* < 0.05) (Figure S3). These findings suggest that the ABO blood type status could be used as a predictor for immuno-chronotherapy efficacy.

We further formally tested the interaction between blood type and infusion timing by including an interaction term in the multivariable Cox regression model. The interaction term was statistically significant (*p* for interaction < 0.05), indicating that the benefit of morning infusion significantly differs between type O and non-type O patients (Tables S4 and S5).

## 4. Discussion

Immunotherapy remains one of the most promising treatment strategies for lung cancer patients. Yet, patient response to ICIs is highly variable, highlighting the urgent need to enhance their efficacy in clinical settings [33,34]. Previous analyses have shown that disruption of the circadian rhythm is associated with the development and progression of breast cancer and hepatoma carcinoma [35,36]. In contrast, Janopaul-Naylor et al. found no association between the timing of ICI administration and clinical outcomes in metastatic head and neck squamous cell carcinoma [37]. Furthermore, many large studies did not control for the timing of ICI administration [38,39]. Whether administering ICIs at scheduled intervals in line with the circadian rhythm can influence disease progression remains unclear.

Using one of the largest real-world cohorts of lung cancer patients from the Chinese alpine region, this study addresses a key gap in chrono-immunotherapy. Early-day administration of PD-1/PD-L1 inhibitors, with infusion completed before 13:00, was consistently associated with significantly improved OS and PFS across all cohorts. Moreover, univariate and multivariable Cox regression model analyses revealed that administering PD-1/PD-L1 inhibitors before 13:00 was associated with a lower mortality risk. The similarities observed in treatment outcomes across all cohorts highlight the consistency of ICIs’ timing responses in patients with lung cancer.

Nevertheless, the recent i-TIMES meta-analysis (ELCC 2026) demonstrated the non-inferiority of late (after 12:00) compared with early (before 12:00) ICI administration (HR = 1.039, 95% CI: 0.925–1.168), with median overall survival of 17.3 months and 16.0 months in the early and late groups, respectively. The inconsistency between our findings and those from the i-TIMES investigation may be attributable to disparities in time cutoff definitions, patient cohorts, and study methodologies [40]. Amid these divergent clinical observations, compelling biological evidence underscores the clinical relevance of chrono-immunotherapy strategies.

The suprachiasmatic nucleus (SCN), the primary circadian pacemaker in mammals, regulates the 24-h rhythmic activity of the immune system, including key cells such as dendritic cells (DCs) and T cells [41,42]. In DCs, these rhythms influence antigen presentation and cytokine production, enhancing their ability to activate T cells [43]. In T cells, circadian regulation influences their proliferation, migration, and ability to target tumor cells effectively [44]. Together, these cell-autonomous circadian clocks optimize the immune response, contributing to the body’s anti-tumor activity [45]. Murine tumor implantation at ZT9 (16:00) significantly increases antigen-specific DC and CD8^+^ T-cell expansion in draining lymph nodes compared with ZT21 (04:00) [13]. In humans, the number of circulating T cells at the circadian nadir (14:00) is approximately 40% lower than at the circadian peak (2:00) [46]. Huang et al. demonstrated that the efficacy of anti–PD-1 therapy depends on the ratio of peripheral exhausted T-cell reinvigoration to baseline tumor burden rather than the absolute magnitude of the immune response [47]. Administering PD-1/PD-L1 inhibitors during the ascending phase of this immune cycle, before the afternoon decline in circulating T cells and the rise in suppressive Tregs, may optimize tumor-antigen encounter and immune synapse formation. The 13:00 cutoff was chosen because it is consistent with thresholds reported in several previous retrospective studies [48,49]. It also matches the transitional window of the immune cycle, when circulating T cells are at their daytime peak, and corresponds to the median infusion time during the first four treatment cycles in our cohort. This approach combines biological rationale with real-world clinical practice [50].

Circadian pharmacokinetics also influence the clinical effect of ICIs. Francis et al. showed that following IV infusion, tracer-labeled pembrolizumab distributes well to secondary lymphoid organs, including tumor-draining LNs, where most anti-tumor T-cell activity occurs [51]. Administering ICIs earlier in the day may enhance antibody colocalization with circulating T cells, thereby promoting more effective T-cell activation. Although prospective node-level immune monitoring is lacking, our clinical data indicate that completing ICI infusions before 13:00 is associated with significantly prolonged OS and PFS, consistent with these mechanistic insights.

Additionally, our study evaluated the impact of blood type on treatment outcomes following ICI administration. Compared with patients of non-O blood types (A, B, or AB), those with blood type O experienced a higher incidence of irAEs following immunotherapy and significantly prolonged OS and PFS. Patients with type O blood lack specific surface antigens on their red blood cells, which may broaden their T-cell repertoire. This expanded variety of T cells could allow ICIs to more effectively stimulate an immune response against cancer cells [23]. Over time, tumors may evolve to preferentially select for neoantigens that resemble blood proteins, which are a functional blind spot in the T-cell repertoire [52]. Further investigation into the relationship between ICI infusion and patients’ blood type could help address gaps in understanding the T-cell immune repertoire. Furthermore, a stronger association between survival and the timing of ICI infusion was observed in female patients compared to males in our study. This difference may be attributed to sex-related variations in circadian rhythms and the body’s response to chrono-therapy [16]. Overall, these findings highlight the need for further mechanistic investigations to optimize the administration of ICIs.

Recent studies have reported a significantly higher incidence of mild-to-moderate skin reactions in patients receiving morning ICI infusions compared with those receiving afternoon infusions. Conversely, morning administration resulted in a better tumor immune response and was associated with significantly less moderate-to-severe fatigue [53,54]. In our research, irAEs were significantly correlated with PFS. Compared to injections after 13:00, those administered before 13:00 seem to have a lower incidence of irAEs. These findings underscore the potential importance of circadian timing in optimizing immunotherapy outcomes.

This study has several limitations that have to be acknowledged. Its retrospective design may have led to variation in ICI administration across patients, potentially affecting the immune response. In addition, individual differences in circadian rhythms may have also introduced an additional confounding variable. Dichotomizing patients by a non-standardized cutoff (before versus after 13:00) further limits the precision of the analysis and may obscure true circadian effects. The use of different ICIs adds heterogeneity in PK and PD profiles, which could influence outcomes. These limitations underscore the need for well-designed prospective studies. Trials using a single ICI with fixed administration times would be particularly valuable for isolating the effect of infusion timing on clinical outcomes.

## 5. Conclusions

Overall, our study provides compelling real-world evidence that administering immunotherapy before 13:00 is associated with a significantly improved prognosis for patients with lung cancer in the Chinese alpine region. Our findings highlight the importance of circadian rhythms in influencing treatment outcomes with immunotherapy. Although prospective studies on the timing of ICIs are warranted, efforts towards scheduling infusions before 13:00 could be considered to improve outcomes in lung cancer patients.

## Figures and Tables

**Table 1:** Clinical characteristics of the patients in Cohort 1, Cohort 2, and the pooled Cohort.

Characteristics	Overall (*n* = 1247)	Cohort 1 (*n* = 839)	Cohort 2 (*n* = 408)	*p*-Value (Cohort 1 vs. 2)
Sex				0.421
Male	849 (68.1%)	565 (67.3%)	284 (69.6%)	
Female	398 (31.9%)	274 (32.7%)	124 (30.4%)	
Age				0.983
≤60	571 (45.8%)	384 (45.8%)	187 (45.8%)	
>60	676 (54.2%)	455 (54.2%)	221 (54.2%)	
Smoking Status				0.003
Yes	694 (55.7%)	491 (58.5%)	203 (49.8%)	
No	553 (44.3%)	348 (41.5%)	205 (50.2%)	
Histological type				<0.01
Adenocarcinoma	533 (42.7%)	316 (37.7%)	217 (53.2%)	
Squamous cell carcinoma	306 (24.6%)	178 (21.2%)	128 (31.4%)	
Small cell lung cancer	408 (32.7%)	345 (41.1%)	63 (15.4%)	
Tumor location				0.233
Central-type	568 (45.5%)	392 (46.7%)	176 (43.1%)	
Peripheral-type	679 (54.5%)	447 (53.3%)	232 (56.9%)	
Blood type				0.818
Type O blood	523 (41.9%)	350 (41.7%)	173 (42.4%)	
Type A/B/AB blood	724 (58.1%)	489 (58.3%)	235 (57.6%)	
Family history of cancer				0.029
Yes	317 (25.4%)	229 (27.3%)	88 (21.6%)	
No	930 (74.6%)	610 (72.7%)	320 (78.4%)	
Stage				0.062
III	405 (32.5%)	258 (30.8%)	147 (36.0%)	
IV	842 (67.5%)	581 (69.2%)	261 (64.0%)	
Main sites of metastases				
Brain	262 (21.0%)	199 (23.7%)	63 (15.4%)	<0.01
Liver	188 (15.1%)	140 (16.6%)	48 (11.8%)	0.023
Bone	394 (31.6%)	276 (38.2%)	118 (28.9%)	0.157
Pleural/pericardial metastasis	458 (36.7%)	311 (37.1%)	147 (36.0%)	0.721
Adrenal metastasis	111 (8.9%)	78 (9.2%)	33 (8.0%)	0.482
Number of metastatic sites				0.004
1	405 (32.5%)	260 (30.1%)	145 (35.5%)	
2	340 (27.3%)	244 (29.1%)	96 (23.5%)	
≥3	100 (8.0%)	79 (9.4%)	21 (5.1%)	
IrAEs				0.743
Yes	368 (29.5%)	255 (26.8%)	113 (27.7%)	
No	909 (72.9%)	614 (73.2%)	295 (72.3%)	
PD-L1 tumor proportion score				<0.01
<1%	277 (31.8%)	185 (33.1%)	92 (29.3%)	
1–49%	421 (48.2%)	281 (50.4%)	140 (44.6%)	
≥50%	174 (20.0%)	92 (16.5%)	82 (26.1%)	
Missing	375	281	94	
Median time-of-day when the first four ICI infusions were administered per patient				0.012
Median [IQR]	13:10 [12:11–14:22]	12:58 [11:53–14:26]	13:20 [12:31–14:14]	

Data were presented as median (interquartile range) or n (%). IQR: Interquartile range; IrAEs: Immune-related adverse events.

**Table 2:** Main patient characteristics in the pooled cohort were dichotomized based on the administration of the initial four immunotherapy infusions, with infusions given before or after 13:00.

Characteristics	Before 13:00 (*n* = 662)	After 13:00 (*n* = 585)	*p*-Value
Sex			0.835
Male	449 (67.8%)	400 (68.4%)	
Female	213 (32.2%)	185 (31.6%)	
Age			0.659
≤60	307 (46.4%)	264 (45.1%)	
>60	355 (53.6%)	321 (54.9%)	
Smoking Status			0.007
Yes	345 (52.1%)	349 (59.7%)	
No	317 (47.9%)	236 (40.3%)	
Histological type			0.023
Adenocarcinoma	305 (46.1%)	228 (39.0%)	
Squamous cell carcinoma	160 (24.2%)	146 (25.0%)	
Small cell lung cancer	197 (29.7%)	211 (36.0%)	
Tumor location			0.605
Central-type	297 (44.9%)	271 (46.3%)	
Peripheral-type	365 (55.1%)	314 (53.7%)	
Blood type			<0.001
Type O blood	315 (47.6%)	208 (35.6%)	
Type A/B/AB blood	347 (52.4%)	377 (64.4%)	
Family history of cancer			0.024
Yes	151 (22.8%)	166 (28.4%)	
No	511 (77.2%)	419 (71.6%)	
Stage			0.808
III	213 (32.2%)	192 (32.8%)	
IV	449 (67.8%)	393 (67.2%)	
Main sites or metastases			
Brain	126 (19.0%)	136 (23.2%)	0.068
Liver	101 (15.2%)	87 (14.9%)	0.850
Bone	198 (29.9%)	196 (33.5%)	0.173
Pleural/pericardial metastases	252 (37.7%)	208 (35.6%)	0.419
Adrenal metastases	64 (9.6%)	47 (8.0%)	0.312
Number of metastatic sites			0.464
1	225 (34.0%)	180 (30.8%)	
2	179 (27.0%)	161 (27.5%)	
≥3	47 (7.1%)	53 (9.1%)	
IrAEs			0.956
Yes	179 (27.0%)	159 (27.2%)	
No	483 (73.0%)	426 (72.8%)	
ECOG performance status			0.285
0	366 (55.3%)	342 (58.5%)	
1	296 (44.7%)	244 (41.7%)	
LDH concentration before treatment			0.734
≤280 U/L	475 (71.8%)	420 (71.8%)	
>280 U/L	181 (28.2%)	165 (28.2%)	
Immune checkpoint inhibitor regimen administered			0.534
PD-1	385 (58.2%)	330 (56.4%)	
PD-L1	277 (41.8%)	255 (43.6%)	
PD-L1 tumor proportion score			0.022
<1%	136 (28.4%)	141 (35.9%)	
1–49%	241 (50.3%)	180 (45.8%)	
≥50%	102 (21.3%)	72 (18.3%)	
Missing	183	192	

Data were presented as median (interquartile range) or *n* (%); IrAEs: Immune-related adverse events.

**Table 3:** Relationship between clinical response and time-of-day administration in each cohort.

Response	Cohort 1			Cohort 2			Pooled Cohort		
	Before (*n* = 430)	After (*n* = 409)	*p*-Value	Before (*n* = 232)	After (*n* = 176)	*p*-Value	Before (*n* = 662)	After (*n* = 585)	*p*-Value
DCR			<0.01			0.009			<0.01
CR+PR+SD	363 (84.4%)	300 (73.3%)		202 (87.1%)	136 (77.3%)		565 (85.3%)	436 (74.5%)	
PD	67 (15.6%)	109 (26.7%)		30 (12.9%)	40 (22.7%)		97 (14.7%)	149 (25.5%)	
ORR			0.005 *			0.098			<0.01
CR+PR	277 (64.4%)	225 (55.0%)		168 (72.4%)	114 (64.8%)		445 (67.2%)	339 (57.9%)	
SD+PD	153 (35.6%)	184 (45.0%)		64 (27.6%)	62 (35.2%)		217 (32.8%)	246 (42.1%)	

* Indicating statistically significant difference after Bonferroni correction for multiple comparisons. PR: Partial response; CR: Complete response; PD: Progressive disease; SD: Stable disease. The DCR was defined as the sum of PR, CR, and SD; The ORR was defined as the sum of CR and PR.

**Table 4:** Association of the stratified blood type with irAEs development.

Blood Type	IrAEs *n* (%)	*p*-Value	Univariate		Multivariate	
			OR (95% CI)	*p*-Value	OR (95% CI)	*p*-Value
O (*n* = 523)	186 (35.6)		1		1	
A/B/AB (*n* = 724)	152 (20.1)	<0.001	0.481 (0.374–0.620)	<0.01	0.486 (0.377–0.627)	<0.01

Multivariate logistic regression analysis was adjusted for sex, age, smoking status, and family history of cancer as covariates. OR: Odds ratio; CI: Confidence interval; IrAEs: Immune-related adverse events.

## Data Availability

The data that support the findings of this study are available on request from the corresponding author. The data are not publicly available due to privacy or ethical restrictions.

## Supplementary Materials

The following supporting information can be downloaded at: https://www.mdpi.com/article/10.3390/cancers18091469/s1. Figure S1: Treatment outcomes of patients with different time-of-day immunotherapy infusions. Kaplan–Meier curves analysis of OS (A) and PFS (B) in the NSCLC, OS (C) and PFS (D) in the SCLC. *p*-values were calculated using the log-rank test. Figure S2: The therapeutic effects of immunotherapy infusion in patients with NSCLC at different times of day. Kaplan–Meier curves analysis of OS (A) and PFS (B) in the TPS < 1%, OS (C) and PFS (D) in the TPS ≥ 1%. *p*-values were calculated using the log-rank test. Figure S3: Treatment outcomes of different blood type patients with time-of-day immunotherapy infusions. Kaplan–Meier curves analysis of OS (A) and PFS (B) in the NSCLC, OS (C) and PFS (D) in the SCLC. *p*-values were calculated using the log-rank test. Table S1: Correlation analysis of baseline characteristics and time-of-day dosing of ICIs (Before vs. After) among cohort populations in the DCR. Table S2: Correlation analysis of baseline characteristics and time-of-day dosing of ICIs (Before vs. After) among cohort populations in the ORR. Table S3: Univariate Cox regression analyses of factors associated with OS and PFS in the pooled cohort. Table S4: The interaction between blood type and infusion timing for OS. Table S5: The interaction between blood type and infusion timing for PFS.

## Abbreviations

The following abbreviations are used in this manuscript:

95% CI	95% confidence interval
CT	Computed tomography
CTCAE	Common terminology criteria for adverse events
CR	Complete response
DC	Dendritic cell
DCR	Disease control rate
ECOG	Eastern Cooperative Oncology Group
HR	Hazard ratio
ICIs	Immune checkpoint inhibitors
IQRs	Interquartile ranges
IrAEs	Immune-related adverse events
LUAD	Lung adenocarcinoma
LUSC	Lung squamous cell carcinoma
LDH	Lactate dehydrogenase
MRI	Magnetic resonance imaging
NSCLC	Non-small cell lung cancer
OR	Odds ratio
ORR	Objective response rate
OS	Overall survival
PET	Positron emission tomography
PD	Progressive disease
PD-1	Programmed death 1
PD-L1	Programmed death-ligand 1
PFS	Progression-free survival
PK	Pharmacokinetics
PR	Partial response
RCSs	Restricted cubic splines
RECIST	Response evaluation criteria in solid tumors
SCLC	Small-cell lung cancer
SCN	The suprachiasmatic nucleus
SD	Stable disease
TPS	Tumor Proportion Score
