# Supplementary material for: Impact of Blood Type and Administration Timing on Therapeutic Outcomes of Immune Checkpoint Inhibitors for Patients with Lung Cancer in the Chinese Alpine Region
Source: Cancers (Basel). 2026 May 2;18(9):1469. doi: 10.3390/cancers18091469 (PMC13162769; doi:10.3390/cancers18091469)
Supplement: Supplementary file 1 [file cancers-18-01469-s001.zip › cancers-4244788-supplementary.pdf]

Supplementary Materials: Impact of Blood Type and Administration Timing on Therapeutic Outcomes of Immune Checkpoint Inhibitors for Patients with Lung Cancer in the Chinese Alpine Region

Meiling Zhang, Xin Zhang, Tie Lin, Bao Liu, Jingwei Hao, Ziyi Gao, Xiaoli Li and Meng Wang

Table S1. Correlation analysis of baseline characteristics and time-of-day dosing of ICIs (Before vs. After) among cohort populations in the DCR.

Characteristics	Cohort 1 (DCR)			Cohort 2 (DCR)			Pooled cohort (DCR)		
	Before (n=363)	After (n=300)	*p*-Value	Before (n= 202)	After (n=136)	*p*-Value	Before (n=565)	After (n=436)	*p*-Value
**Age**			0.406			0.056			0.076
≤60	169	130		100	53		269	183	
>60	194	170		102	83		296	253	
**Sex**			0.674			0.764			0.811
Male	244	197		141	97		385	294	
Female	119	103		61	39		180	142	
**Smoking Statue**			0.060			0.084			0.008
Yes	200	187		95	77		295	264	
No	163	113		107	59		270	172	
**Blood type**			<0.001			0.041			< 0.001
Type O blood	178	109		97	50		275	159	
Type A/B/AB blood	185	191		105	86		290	277	
**Family history of cancer**			0.989			0.603			0.691
Yes	93	77		37	28		130	105	
No	270	223		165	108		435	311	

The DCR was defined as the sum of PR, CR, and SD.

Table S2. Correlation analysis of baseline characteristics and time-of-day dosing of ICIs (Before vs. After) among cohort populations in the ORR.

Characteristics	Cohort 1 (ORR)			Cohort 2 (ORR)			Pooled cohort (ORR)		
	Before (n=277)	After (n=225)	*p*-Value	Before (n= 168)	After (n=114)	*p*-Value	Before (n=445)	After (n=339)	*p*-Value
**Age**			0.248			0.091			0.051
≤60	130	94		85	46		215	140	
>60	147	131		83	68		230	199	
**Sex**			0.477			0.927			0.570
Male	180	153		120	82		300	235	
Female	97	72		48	32		145	104	
**Smoking Statue**			0.050			0.109			0.009
Yes	147	139		78	64		228	133	
No	130	86		90	50		217	206	
**Blood type**			0.004			0.91			<0.001
Type O blood	143	87		85	46		228	133	
Type A/B/AB blood	134	138		83	68		217	206	
**Family history of cancer**			0.737			0.802			0.602

Yes	69	59	29	21	98	80
No	208	166	139	93	347	259

The ORR was defined as the sum of CR and PR.

Table S3. Univariate Cox regression analyses of factors associated with OS and PFS in the pooled cohort.

Characteristics	OS (Univariate)		PFS (Univariate)	
	HR (95% CI)	P-value	HR (95% CI)	P-value
**Time-of-day**		< 0.001		< 0.001
Before 13:00	Reference		reference	
After 13:00	1.95 (1.575-2.328)		1.598 (1.380-1.852)	
**Age**		0.347		0.318
≤60	Reference		reference	
>60	0.912 (0.735-1.105)		1.076 (0.932-1.244)	
**Sex**		0.204		0.799
Female	Reference		Reference	
Male	1.143 (0.930-1.405)		1.020 (0.875-1.190)	
**Family history of cancer**		0.165		0.142
No	Reference		Reference	
Yes	0.850 (0.676-1.609)		0.879 (0.740-1.004)	
**Smoking**		0.092		0.78
No	Reference		Reference	
Yes	1.180 (0.973-1.430)		0.980 (0.848-1.132)	
**Histological type**		0.392		< 0.001
LUAD	Reference		Reference	
LUSC	0.875 (0.609-1.144)	0.392	0.783 (0.606-0.898)	0.002
SCLC	1.804 (1.458-2.232)	< 0.01	1.546 (1.316-1.816)	< 0.001
**Tumor location**		0.534		0.136
Central	Reference		Reference	
Perpheral	0.941 (0.777-1.140)		0.896 (0.776-1.035)	
**Blood type**		< 0.001		< 0.001
O type	Reference		Reference	
Non-O type	1.686 (1.383-2.055)		1.452 (1.251-1.685)	
**Stage**		< 0.001		< 0.001
III	Reference		Reference	
IV	2.760 (2.162-3.524)		2.223 (1.883-2.625)	
**Brain metastatic**		< 0.001		< 0.001
No	Reference		Reference	
Yes	4.113 (3.365-5.028)		2.909 (2.475-3.420)	
**Liver metastatic**		< 0.001		< 0.001
No	Reference		Reference	
Yes	1.869 (1.481-2.359)		1.646 (1.366-1.984)	
**Bone metastatic**		< 0.001		< 0.001
No	Reference		Reference	
Yes	2.969 (2.449-3.599)		1.965 (1.691-2.283)	
**Pleural metastatic**		0.475		< 0.001
No	Reference		Reference	
Yes	1.075 (0.882-1.310)		1.326 (1.145-1.537)	
**Adrenal metastatic**		< 0.001		< 0.001
No	Reference		Reference	
Yes	2.448 (1.874-3.198)		1.885 (1.507-2.359)	
**Number of Metastatic sites**		< 0.001		< 0.001
0	Reference		Reference	
1	1.184 (0.869-1.613)	0.285	1.477 (1.189-2.956)	< 0.001

2	4.329 (3.330-5.627)	< 0.001	2.996 (2.481-3.691)	< 0.001
3	7.233 (5.213-10.035)	< 0.001	4.425 (3.420-5.727)	< 0.001
**IRAEs**		0.046		0.511
No	Reference		Reference	
Yes	0.807 (0.654-0.996)		1.503 (0.902-1.230)	
**PD-L1**		< 0.001		< 0.001
Missing	Reference		Reference	
< 1%	2.804 (1.648-2.636)	< 0.001	2.382 (1.923-2.829)	< 0.001
1% - 49%	0.506 (0.389-0.657)	< 0.001	0.588 (0.488-0.710)	
≥ 50%	0.252 (0.174-0364)	< 0.001	0.473 (0.371-0.603)	
**ICIs**		0.068		0.292
PD-1	Reference		Reference	
PD-L1	0.834 (0.686-1.014)		0.925 (0.799-1.070)	
**LDH**		0.103		0.211
> 280	Reference		Reference	
≤ 280	0.842 (0.685-1.035)		0.967 (0.837-1.119)	
**ECOG**		0.270		0.655
0	Reference		Reference	
1	1.114 (0.92-1.348)		0.967 (0.837-1.197)	

Table S4. The interaction between blood type and infusion timing for OS.

Characteristics	HR (95% CI)	*p*-Value
**Time of day infusion**		< 0.001
Before 13:00	Reference	
After 13:00	1.874 (1.376-2.552)	
**Blood type**		< 0.001
O blood	Reference	
Non-O blood	1.624 (1.238-2.131)	
***P* for Interaction (Time of day infusion*blood type)**		0.048

The primary endpoints of OS.

Table S5. The interaction between blood type and infusion timing for PFS.

Characteristics	HR (95% CI)	*p*-Value
**Time of day infusion**		< 0.001
Before 13:00	Reference	
After 13:00	1.525 (1.215-1.913)	
**Blood type**		0.002
O blood	Reference	
Non-O blood	1.380 (1.128-1.688)	
***P* for Interaction (Time of day infusion*blood type)**		0.02

The primary endpoints of PFS.

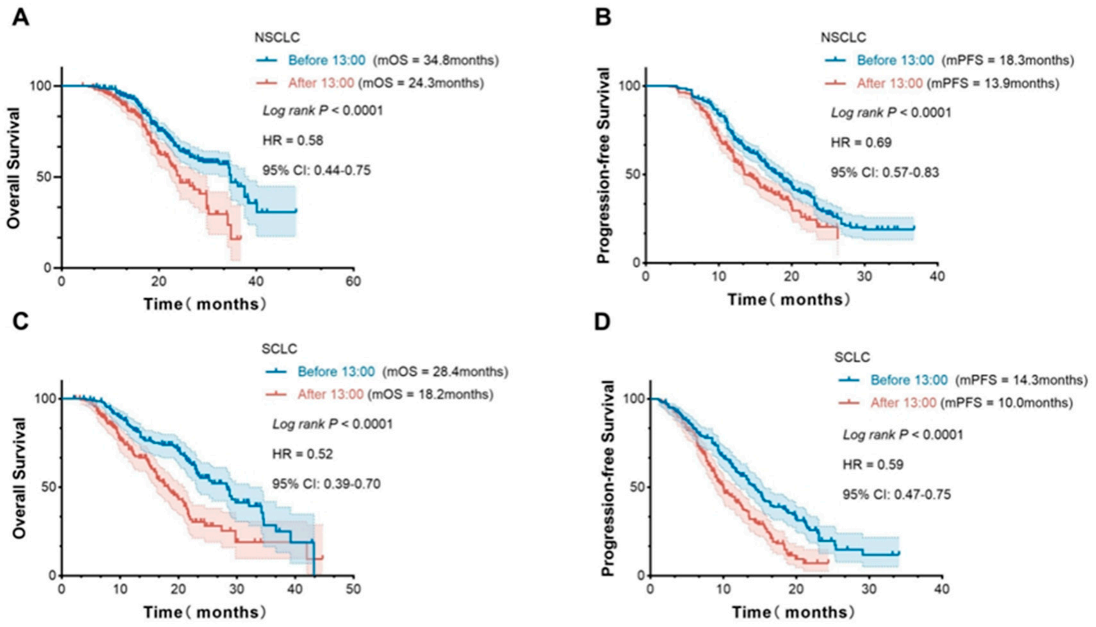

Figure S1. Treatment outcomes of patients with different time-of-day immunotherapy infusions. Kaplan-Meier curves analysis of OS (**A**) and PFS (**B**) in the NSCLC, OS (**C**) and PFS (**D**) in the SCLC. *P*-values were calculated using the log-rank test.

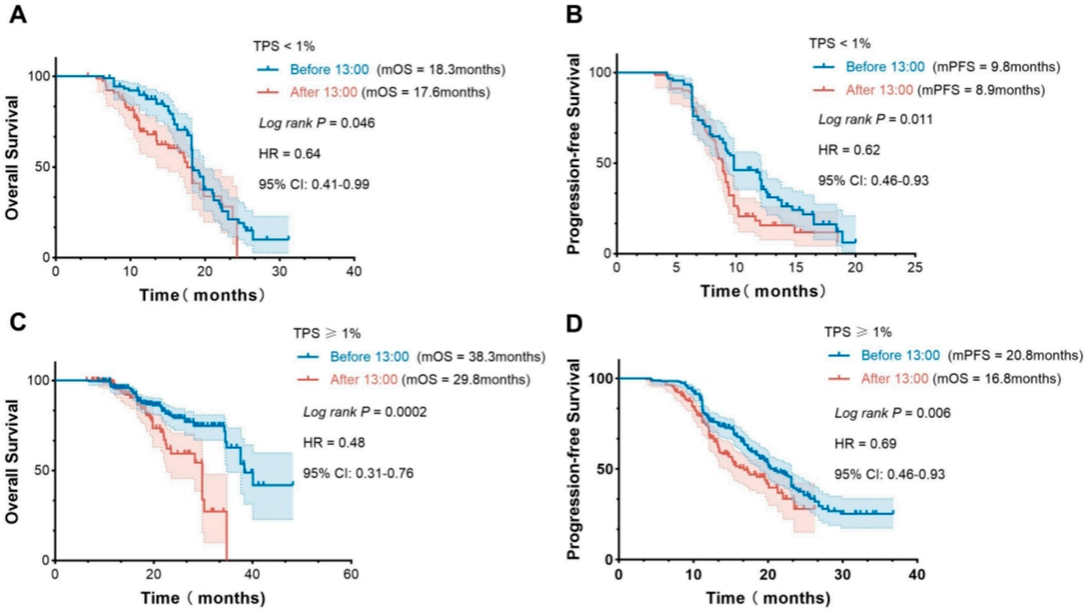

Figure S2. The therapeutic effects of immunotherapy infusion in patients with NSCLC at different times of day. Kaplan-Meier curves analysis of OS (**A**) and PFS (**B**) in the TPS < 1%, OS (**C**) and PFS (**D**) in the TPS ≥ 1%. *P*-values were calculated using the log-rank test.

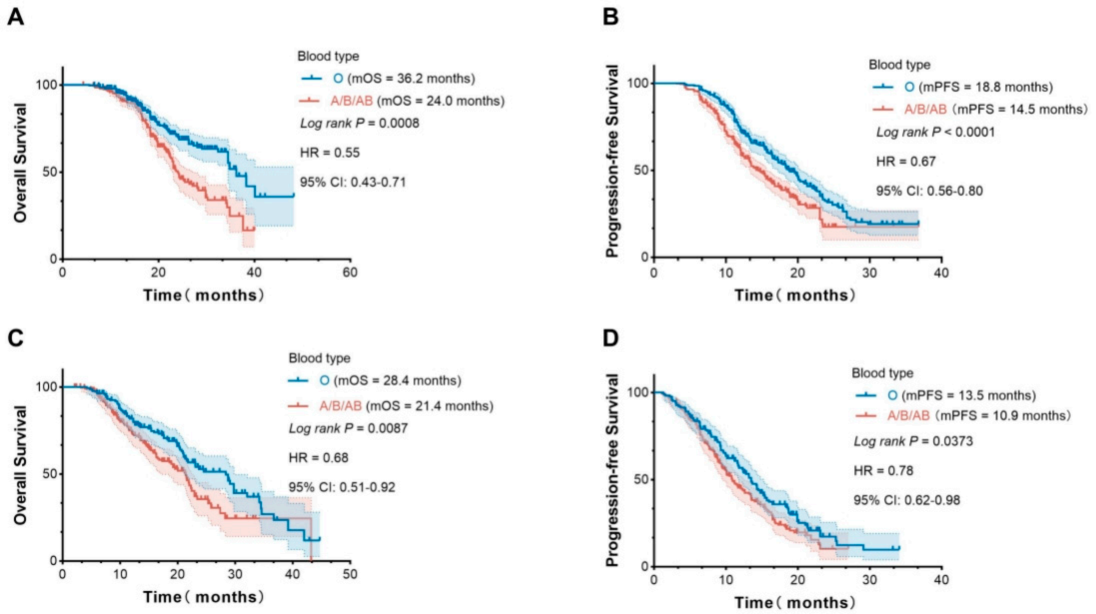

Figure S3. Treatment outcomes of different blood type patients with time-of-day immunotherapy infusions. Kaplan-Meier curves analysis of OS (**A**) and PFS (**B**) in the NSCLC, OS (**C**) and PFS (**D**) in the SCLC. *P*-values were calculated using the log-rank test.
